# Trachoma Prevalence and Associated Risk Factors in The Gambia and Tanzania: Baseline Results of a Cluster Randomised Controlled Trial

**DOI:** 10.1371/journal.pntd.0000861

**Published:** 2010-11-02

**Authors:** Emma M. Harding-Esch, Tansy Edwards, Harran Mkocha, Beatriz Munoz, Martin J. Holland, Sarah E. Burr, Ansumana Sillah, Charlotte A. Gaydos, Dianne Stare, David C. W. Mabey, Robin L. Bailey, Sheila K. West, for the PRET Partnership

**Affiliations:** 1 Clinical Research Unit, Department of Infectious and Tropical Diseases, London School of Hygiene & Tropical Medicine, London, United Kingdom; 2 Kongwa Trachoma Project, Kongwa, Tanzania; 3 Dana Center for Preventive Ophthalmology, Wilmer Eye Institute, Johns Hopkins University, Baltimore, Maryland, United States of America; 4 Medical Research Council Laboratories, Fajara, Banjul, The Gambia; 5 National Eye Care Programme, Gambian Department of State for Health and Social Welfare, Banjul, The Gambia; 6 Division of Infectious Diseases, Medicine, Johns Hopkins Medical Institutions, Baltimore, Maryland, United States of America; University of California San Francisco, United States of America

## Abstract

**Background:**

Blinding trachoma, caused by ocular infection with *Chlamydia trachomatis*, is targeted for global elimination by 2020. Knowledge of risk factors can help target control interventions.

**Methodology/Principal Findings:**

As part of a cluster randomised controlled trial, we assessed the baseline prevalence of, and risk factors for, active trachoma and ocular *C. trachomatis* infection in randomly selected children aged 0–5 years from 48 Gambian and 36 Tanzanian communities. Both children's eyes were examined according to the World Health Organization (WHO) simplified grading system, and an ocular swab was taken from each child's right eye and processed by Amplicor polymerase chain reaction to test for the presence of *C. trachomatis* DNA. Prevalence of active trachoma was 6.7% (335/5033) in The Gambia and 32.3% (1008/3122) in Tanzania. The countries' corresponding Amplicor positive prevalences were 0.8% and 21.9%. After adjustment, risk factors for follicular trachoma (TF) in both countries were ocular or nasal discharge, a low level of household head education, and being aged ≥1 year. Additional risk factors in Tanzania were flies on the child's face, being Amplicor positive, and crowding (the number of children per household). The risk factors for being Amplicor positive in Tanzania were similar to those for TF, with the exclusion of flies and crowding. In The Gambia, only ocular discharge was associated with being Amplicor positive.

**Conclusions/Significance:**

These results indicate that although the prevalence of active trachoma and Amplicor positives were very different between the two countries, the risk factors for active trachoma were similar but those for being Amplicor positive were different. The lack of an association between being Amplicor positive and TF in The Gambia highlights the poor correlation between the presence of trachoma clinical signs and evidence of *C. trachomatis* infection in this setting. Only ocular discharge was associated with evidence of *C. trachomatis* DNA in The Gambia, suggesting that at this low endemicity, this may be the most important risk factor.

**Trial Registration:**

ClinicalTrials.gov NCT00792922

## Author Summary

Trachoma is caused by *Chlamydia trachomatis* and is the leading infectious cause of blindness. The World Health Organization's (WHO) control strategy includes antibiotic treatment of all community members, facial cleanliness, and environmental improvements. By determining how prevalent trachoma is, decisions can be made whether control activities need to be put in place. Knowing what factors make people more at risk of having trachoma can help target trachoma control efforts to those most at risk. We looked at the prevalence of active trachoma and *C. trachomatis* infection in the eyes of children aged 0–5 years in The Gambia and Tanzania. We also measured risk factors associated with having active trachoma or infection. The prevalence of both active trachoma and infection was lower in The Gambia (6.7% and 0.8%, respectively) than in Tanzania (32.3% and 21.9%, respectively). Risk factors for active trachoma were similar in the two countries. For infection, the risk factors in Tanzania were similar to those for TF, whereas in The Gambia, only ocular discharge was associated with infection. These results show that although the prevalence of active trachoma and infection is very different between the two countries, the risk factors for active trachoma are similar but those for infection are different.

## Introduction

Trachoma is caused by ocular infection with serovars A, B, Ba or C of the bacterium *Chlamydia trachomatis*. It is the leading infectious cause of blindness worldwide [1] with an estimated 40.6 million people suffering from active trachoma (trachomatous inflammation, follicular (TF) and/or intense (TI)) and 8.2 million having trichiasis [2]. As part of the “SAFE” (**S**urgery, **A**ntibiotics, **F**acial cleanliness, **E**nvironmental improvement) trachoma control strategy, the World Health Organization (WHO) recommends mass antibiotic treatment annually for at least three years of all individuals in any district or community where the prevalence of TF in children aged 1–9 years is at least 10%. After three or more years of A, F and E interventions, the prevalence is reassessed and a decision is made regarding the need to continue or cease treatment [3]. Mass antibiotic treatment aims to clear infection from the community, most of which is found in children [4].

Trachoma is endemic in both The Gambia and Tanzania, with estimated active trachoma prevalences in children aged 1–9 years of 10.4% and 27%, respectively [5], [6]. Accordingly, they have both recently qualified for a donation of the antibiotic azithromycin for mass treatment by Pfizer via the International Trachoma Initiative. Given the different endemicities of these two countries, one in which trachoma is almost disappearing and one in which trachoma shows only modest signs of being reduced, the question of whether the same risk factors are predictive of trachoma is of interest. In addition, since the presence of trachoma clinical signs is often poorly correlated with that of ocular *C. trachomatis* infection [5], [7], [8], [9], [10], [11], the risk factors for these markers of trachoma may also differ.

Studies have shown that although young age is a common risk factor for active trachoma, other risk factors may be setting-specific. Furthermore, few studies have simultaneously reported risk factors for active trachoma and ocular *C. trachomatis* infection within the same setting [12], [13]. Information on risk factors can contribute to our understanding of trachoma transmission within the study area, and the targeting of trachoma control interventions can be aided through knowledge of risk factors.

We aimed to assess the prevalence of, and risk factors for, both active trachoma and ocular *C. trachomatis* infection pre-treatment in The Gambia and Tanzania, as part of the Partnership for the Rapid Elimination of Trachoma (PRET) cluster randomised controlled trial. The aims of PRET are to test the impact on the prevalence of active trachoma and ocular *C. trachomatis* infection, as detected by Amplicor PCR, after three years in communities mesoendemic for trachoma (between 20% and 50% TF) or hypoendemic (between 10% and 20% TF), when communities are randomised to different mass treatment population coverage levels and a different number of rounds of treatment, with a graduation rule if the prevalence of TF or detected ocular *C. trachomatis* infection falls below 5% (Stare *et al.* submitted).

The data presented here are from the baseline surveys of PRET, where data on the prevalence of TF and evidence of ocular *C. trachomatis* infection were collected, and risk factors for these outcomes were obtained in a standardised fashion.

## Methods

The study methods have been described in detail elsewhere (Stare *et al.* submitted) and are summarised below. Reporting of the study has been verified in accordance with the STROBE (Strengthening the Reporting of Observational Studies in Epidemiology) checklist (provided as supporting information, Checklist S1).

### Ethical approval

The research was done in accordance with the declaration of Helsinki. Ethical approval was obtained from the London School of Hygiene & Tropical Medicine (LSHTM), UK, Ethics Committee; The Gambia government/Medical Research Council (MRC) Joint Ethics Committee, The Gambia; the Johns Hopkins Institutional Review Board; and the Tanzanian National Institute for Medical Research. Oral consent was obtained from the village leaders, and written (thumbprint or signature) consent from the child's guardian at the time of examination, which was signed by an independent witness.

### Site selection

**Figure 1:**
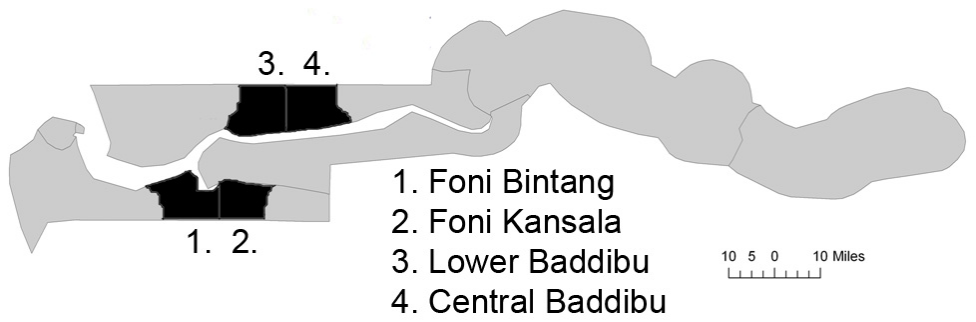
Location of study districts in The Gambia.

**Figure 2:**
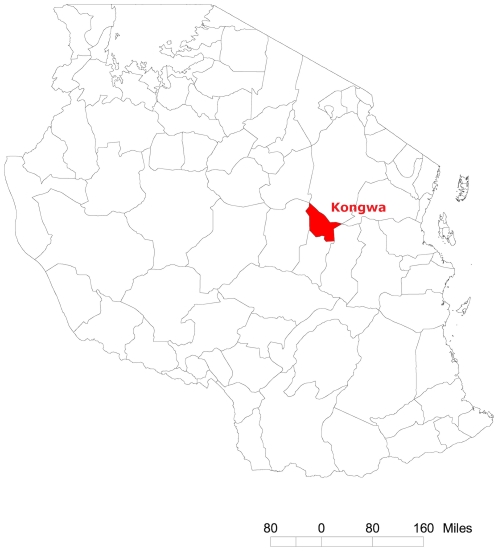
Location of Kongwa district in Tanzania.

In The Gambia, 48 census Enumeration Areas (EAs), designed to have similar population sizes of between 600–800 people, were randomly selected from within 4 strata consisting of the following districts: Foni Bintang and Foni Kansala in Western Region, and Central Baddibu and Lower Baddibu in North Bank Region (12 EAs per district) (Figure 1). In Tanzania, 32 communities (geographically distinct areas within a village with an average population of approximately 1500 people) were selected in Kongwa district, Dodoma region (Figure 2). Tanzanian communities were selected based on having an active trachoma prevalence above 20% in preliminary surveys and were therefore not randomly selected as they were in The Gambia.

### Data collection

A week-long workshop was conducted in February 2008 to standardise all fieldwork methods, including trachoma grading, photography, sample collection, form filling, facial cleanliness status grading, and data entry. For trachoma grading, graders were standardised against a senior grader (RB) every day by examining participants in the field. A kappa of >0.6 for TF grading was required between the senior grader and the graders in the final grading exam. All other procedures had to be performed correctly five times in the field under observation by senior investigators before certification was given. Fieldwork in The Gambia took place between 19^th^ May 2008 and 29^th^ July 2008. In Tanzania, data collection was between 15^th^ May and 1^st^ November 2008.

#### Census

Census collection differed between the two countries as a result of high population movement within The Gambia, which is not such a problem in Kongwa district, Tanzania. In The Gambia a census was made of the *de facto* population (those who had slept in the household the night before) within the week before examination to limit absenteeism. In Tanzania the census included all persons resident in the village for at least three months unless newly arrived or newborn. In both countries, the census served as the basis for making a random selection of 100 sentinel children aged 0–5 years in each community for examination.

#### Examination

After obtaining consent, both upper eyelids of the sentinel children were graded for active trachoma (TF and/or TI) according to the WHO simplified grading system [14], using a 2.5× magnifying loupe and adequate sun or torch light. Two photographs were taken from the right eye of all children in The Gambia, and a random selection of children in Tanzania, using a Nikon D-series camera and a Micro Nikon 105mm 1∶2.8G lens (Nikon, Tokyo, Japan). Photographs were used for grading quality assurance after field data collection whereby a set of 50 photos per grader was graded by the senior grader and the field examiners. A kappa score of at least 0.6 between the examiners' and senior grader's TF photo grading was required for the examiner to pass the quality control exercise.

A fieldworker then passed a dacron swab (MDCI Ltd, Crawley, UK, for The Gambia; Fisher Healthcare, Houston, TX, for Tanzania) to the grader who took one swab from each child's right eye. The swab was held parallel to the conjunctiva and passed three times with enough pressure to cause blanching, with a rotation of 120° between each pass. The grader placed the swab in a screw-top tube (VWR, Lutterworth, UK, for The Gambia; Sarstedt AG & Co, Numbrecht, Germany, for Tanzania) and snapped the swab's stem. The tube was labelled with a unique ID number, placed in a racked box in a cooler, and frozen within 10 hours. The grader changed gloves between each child to limit cross-contamination. The fieldworker changed gloves when any potential contamination of the gloves was thought to have occurred.

For every 100 ocular swabs, at least three air controls were taken at random. Immediately after the ocular swab was taken, a new swab was passed 5 cm from the child's everted right eyelid five times, and labelled so that the control appeared as a genuine sample to the laboratory staff. To further validate the laboratory results and ensure standardisation between laboratories, a set of 20 mixed positive and negative samples were sent from each laboratory to the external laboratory at the University of California in San Francisco (UCSF) for processing. An agreement of 90% was required to pass this validation exercise.

#### Risk factors

Household-level risk factor data were obtained at the time of census using a questionnaire. Questions related to the number of years of household head formal education, access to a latrine, whether the time to fetch water was more than 30 minutes (with reference to a locally appropriate common activity time), and awareness of the presence of a face-washing campaign in the village within the last year that was not a radio or television campaign. Child-level data on facial cleanliness, measured by the presence of ocular discharge (“sleep” on the eyelashes or lids) or nasal discharge (discharge on the nares, cheeks or lips), and whether any flies landed on the face during the time of examination, were collected at the time of examination. All field workers had been standardised during the initial workshop to assess facial cleanliness indicators, and other risk factors, although no measure of inter-observer variability was made.

#### Sample processing

Samples were tested for evidence of *C. trachomatis* DNA by using the Amplicor *Chlamydia trachomatis/Neisseria gonorrhoeae* (CT/NG) Test (Roche Molecular Systems, Indianapolis, IN, USA) according to the manufacturer's instructions, except for sample extraction where previously published protocols were employed for both the Gambian [15] and Tanzanian [16] samples. In The Gambia, samples were tested in pools of five due to the expected low prevalence of infection. Samples from positive or equivocal pools were tested individually to identify positive samples. The Gambian samples were processed at the MRC laboratories, Fajara, The Gambia. The Tanzanian samples were processed individually in the Johns Hopkins International Chlamydia Laboratory, Baltimore, Maryland, USA.

### Statistical analysis

Data were entered into a customised database (MS Access v2007) developed at the Dana Center, Johns Hopkins University. Key fields were double-entered by different entry clerks. Reports of discrepant, missing or query entries were generated in the database and resolved by reference to the forms, or in some cases by return field visits. Further queries of data inconsistencies were produced in statistical packages (Stata v10, STATA Corp., College Station, TX, USA for the Gambian data; SAS v9.2, SAS Institute Inc., Cary, NC, USA for the Tanzanian data) prior to analysis. All queries were verified against the original paper forms. The analyses presented here were conducted using Stata, v10.

Baseline characteristics of household attributes and population size were summarised for both countries. Evidence of variability between communities (clusters) and households was assessed using random effects logistic regression models assuming a 3-level hierarchy to the data structure (community, household and individual) in null regression models.

Univariate associations with TF and ocular *C. trachomatis* infection in children aged 0–5 years were tested using random effects logistic regression, accounting for between-cluster and between-household variation (variance), comparing models with and without covariates using the likelihood ratio test (LRT). Multivariate model building for TF and *C. trachomatis* infection in both countries employed the same stepwise strategy; age and sex were considered *a priori* risk factors and included in all models. Covariates associated with TF or evidence of *C. trachomatis* infection at the 10% significance level in univariate analyses were added in turn (a forward stepwise approach) and covariates retained in the model if the LRT p-value was ≤0.1. In The Gambia, the final multivariate model also adjusted for district to account for sampling stratified by district.

## Results

### Quality control

#### Grading validation

In Tanzania, the two trachoma graders achieved a kappa score on the photo grading against the senior grader for TF of 0.92 and 0.84. In The Gambia, the results for the three graders were 0.81, 0.67, and 0.38. The grader who did not reach the required kappa score of 0.6 had achieved a kappa of 0.70 between their field grade and the senior grader's photo grading. It was noted that a number of the photos were difficult to grade owing to reflected light and/or poor presentation of the lid. The grader was asked to grade a parallel set of baseline photos from The Gambia which were technically more satisfactory, and achieved a kappa score of 0.79.

#### Air controls

In The Gambia, 280 air controls were collected, and one (0.4%) was positive, which was confirmed at both the MRC and UCSF laboratories. This sample was negative when tested for human mitochondrial DNA (mtDNA) using a previously reported method [5], and the corresponding child's sample did contain mtDNA indicating there had not been an exchange between the child's sample and the air control. The child's sample was Amplicor negative and the eye was graded as clinically normal both in the field and on the photograph by the senior grader. The adjacent field and Amplicor plate samples were Amplicor negative.

In Tanzania, 142 air controls were collected and 7 (4.9%) were positive by Amplicor. After reviewing the records, 2 were the result of mis-labelling of the samples (one in the field, one in the laboratory), and the remaining 5 were part of 4 runs that were discarded because of suspected laboratory contamination.

#### Laboratory standardisation

Of 20 mixed positive and negative samples sent to UCSF for re-testing from Tanzania, 17 were concordant, 1 was inhibitory twice and thus considered negative and 2 were discrepant (both positive at JHU but negative at UCSF). This triggered a request for an additional 20 samples to be sent to UCSF, of which 19 were concordant, exceeding the 90% required agreement between the laboratories. JHU also reprocessed the initial set of samples, repeating the same number of freeze-thaw cycles as UCSF, and all re-run samples were concordant with UCSF laboratory testing.

Of the 20 samples sent from The Gambia, all negatives were confirmed negative except for one which was inhibited. Of the positives, 3 were confirmed positive, 2 were inhibited, and 6 were negative. This prompted another 20 samples to be sent to UCSF. Of these, 18 (90%) were concordant. All 12 negatives were confirmed negative, and 6 of the 8 positives were confirmed positive. The remaining 2 samples that were positive in The Gambia tested negative by Amplicor at UCSF.

### Overview of communities and households

In The Gambia, 5033 children aged 0–5 years were examined. In Tanzania, 3198 children were examined but ocular *C. trachomatis* data were missing from 76 of these children. In The Gambia, there were 9 households with missing data for awareness of a village face-washing education programme. In Tanzania, the number of missing values was 5 for household head education, 6 for time to water, 20 for latrine access, and 693 (of which 677 were recorded as “unknown”) for knowledge of a face-washing health education programme.

Community randomisation units were larger in Tanzania than in The Gambia, containing more, smaller households, as seen from the total population size and average household sizes (Table 1), although similar proportions of the total population were children aged under 10 years. Household heads in The Gambia had less formal education than in Tanzania, whereas latrines and water were less easily accessible in Tanzania. Around one third of households in both countries reported awareness of receiving community face-washing health education programmes.

**Table 1:** Baseline characteristics by study site.

	The Gambia	Tanzania
**Communities**	**48**	**32**
**Households**	**3122**	**10011**
No. people/household, median (IQR)	9 (6–14)	4 (3–6)
Household head years of education, median (IQR)	0 (0–0)	0 (0–7)
>30 minutes to water	410 (13.1)	7536 (75.3)
Latrine access	2798 (89.6)	6489 (64.8)
Health education programme^a^	1053 (33.7)	3390 (33.9)
**Total Population**	**33695**	**46634**
Children 0–5 years	7178 (21.3)	11587 (24.8)
Male children 0–5 years	3659 (10.9)	5769 (12.4)

Data are n (%) unless otherwise stated.

a Householders were each asked if they recalled a health education program in their community in the previous year.

### Prevalence of active trachoma and ocular *C. trachomatis* infection

#### The Gambia

A total of 5036 children aged 0–5 years were examined in The Gambia with only 3 missing values for clinical sign data. The overall prevalence of TF in 0–5 year-olds in The Gambia was 6.3% (95% CI: 5.6–7.0). Only 28 (0.6%) children had TI, and 97 (1.9%) had follicles that did not fulfil the WHO simplified grading system criteria for TF (5 or more follicles in the upper tarsal conjunctiva of at least 0·5 mm). Overall, less than 1% of children sampled in The Gambia tested positive by Amplicor. There was a poor correlation between the presence of ocular *C. trachomatis* infection and active trachoma. Indeed, the majority of infected individuals in The Gambia were recorded as being clinically normal (Table 2). Those with both TF and TI were more likely to be Amplicor positive.

**Table 2:** Correlation between disease and ocular *C. trachomatis* infection in children aged 0–5 years.

	The Gambia		Tanzania	
	N	Amplicor positive, n (%)	N	Amplicor positive, n (%)
No trachoma	4698	36 (0.8)	2114	191 (9.0)
TF only	307	2 (0.7)	764	323 (42.3)
TI only	19	0 (0)	45	22 (48.9)
TF & TI	9	1 (11.1)	199	148 (74.4)
**Total**	**5033**	**39 (0.8)**	**3122**	**684 (21.9)**

TF = trachomatous inflammation, follicular; TI = trachomatous inflammation, intense; ocular *C. trachomatous* infection measured by Amplicor.

#### Tanzania

In Tanzania, a total of 3198 children aged 0–5 years were examined, with Amplicor results missing for 76 children. Approximately a third of the examined children had TF (30.9%, 95% CI: 29.3–32.6), and around half of those were Amplicor positive, demonstrating a strong association between TF and evidence of infection, which was not seen in The Gambia (Table 2). A total of 244 (7.8%) children had TI, and as seen in The Gambia, children with both TF and TI were more likely to be Amplicor positive. Of children examined, 15.5% had fewer than 5 follicles and therefore did not fulfil the WHO simplified grading system criteria for TF.

### Risk factors for TF

#### Univariate analysis

There was substantial variation at household and cluster levels in both countries (LRT p<0.001). For example, if the Gambian baseline is regarded as a survey in which EAs are sampled from each district, and then children sampled within each EA, the overall design effect for TF was 3.4. There are data justifying the need for 3-levels in random effects regression models. For TF in The Gambia, the between-household variation was 1.11 (standard error (SE) 0.34) and between-cluster variation 1.10 (SE 0.32). For TF in Tanzania, the between-household variation was 1.31 (SE 0.45) and between-cluster variation was 0.75 (SE 0.17). Amplicor laboratory data in Tanzania between-household variation was 6.23 (SE 1.55) and between-cluster variation was 6.23 (SE 1.55). Similar data could not be generated for The Gambia, where only 39 children were Amplicor positive.

In both The Gambia and Tanzania, increased risk (modelled as odds in logistic regression models) of TF was seen for children with ocular or nasal discharge, and flies on the face, at the 5% level of significance. In both countries, there was a departure from a linear trend in risk of TF with increasing age (LRT p<0.001). Consequently, age was modelled as a categorical variable in multivariate analyses. There was possibly a weak unadjusted effect of increased time to fetch water on TF (p = 0.096) (Table 3). Additional risk factors in Tanzania were being Amplicor positive, not having access to a latrine, and possibly more children aged 0 to 5 years per household (Table 3).

**Table 3:** Univariate TF risk factor analyses in children aged 0–5 years in The Gambia and Tanzania.

		The Gambia				Tanzania			
Characteristic		N	TF, n (%)	OR (95% CI)	p-value^a^	N	TF, n (%)	OR (95% CI)	p-value^a^
**Child level**									
Total	5033	316 (6.3)	-	-	3198	981 (30.7)	-	-	
Age (years)	0	792	13 (1.6)	1	<0.001	561	56 (10.0)	1	<0.001
	1	805	50 (6.2)	4.90 (2.51–9.58)		484	127 (26.2)	4.80 (2.96–7.77)	
	2	801	62 (7.7)	6.64 (3.44–12.8)		578	200 (34.6)	8.64 (5.24–14.3)	
	3	895	65 (7.3)	6.53 (3.39–12.6)		567	230 (40.6)	13.1 (7.80–22.2)	
	4	823	69 (8.4)	7.14 (3.72–13.7)		515	203 (39.4)	12.6 (7.42–21.4)	
	5	917	57 (6.2)	5.65 (2.92–10.9)		493	165 (33.5)	8.52 (5.11–14.2)	
Sex	Female	2272	168 (6.9)	1	0.125	1579	486 (30.8)	1	0.931
	Male	2445	148 (5.7)	0.82 (0.63–1.06)		1619	495 (30.6)	0.99 (0.81–1.21)	
Ocular Discharge	No	4577	231 (5.1)	1	<0.001	2884	829 (28.7)	1	<0.001
	Yes	444	83 (18.7)	4.47 (3.19–6.26)		303	149 (49.2)	2.66 (1.92–3.70)	
Nasal Discharge	No	3369	160 (4.8)	1	<0.001	1649	416 (25.2)	1	<0.001
	Yes	1654	155 (9.4)	2.15 (1.63–2.82)		1537	562 (36.6)	1.99 (1.60–2.47)	
Flies on the face	No	4278	255 (6.0)	1	0.019	3011	887 (29.5)	1	<0.001
	Yes	739	57 (7.7)	1.55 (1.08–2.22)		175	91 (52.0)	2.42 (1.58–3.73)	
Amplicor positive	No	4990	313 (6.3)	1	0.959	2438	492 (20.2)	1	<0.001
	Yes	39	3 (7.7)	1.04 (0.27–4.00)		684	471 (68.9)	14.1 (9.38–21.1)	
**Household level**									
Number of people/household^b^	All residents	-	-	1.00 (0.98–1.02)	0.932	-	-	1.04 (1.00–1.09)	0.061
	Children 0–5	-	-	1.03 (0.97–1.09)	0.348	-	-	1.12 (0.99–1.27)	0.075
Household head education	Gm: NoneTz: 0–6 years	4733	310 (6.6)	1	0.017	1678	567 (33.8)	1	0.001
	Gm: ≥1 yearTz: 7+ years	300	6 (2.0)	0.36 (0.14–0.91)		1518	413 (27.2)	0.70 (0.56–0.87)	
Time to water	<30 minutes	4320	259 (6.0)	1	0.096	738	178 (24.1)	1	0.007
	≥30 minutes	713	57 (8.0)	1.47 (0.94–2.30)		2459	803 (32.7)	1.52 (1.12–2.07)	
Latrine access	No	386	31 (8.0)	1	0.431	1082	381 (35.2)	1	0.012
	Yes	4647	285 (6.1)	0.80 (0.47–1.37)		2114	600 (28.4)	0.75 (0.59–0.94)	
Health education programme^c^	No	3342	185 (5.5)	1	0.926	1860	584 (31.4)	1	0.209
	Yes	1667	128 (7.7)	0.98 (0.70–1.38)		1177	328 (27.9)	0.86 (0.67–1.09)	

TF = trachomatous inflammation, follicular; OR = Odds Ratio; CI = Confidence Interval.

a p-value from likelihood ratio test comparing random effects logistic regression models adjusting for between-household and between-cluster variation with, and without, characteristic of interest.

b variables modelled as continuous measures.

c head of household recall of community health education program.

An association between household head education in Tanzania with decreased risk of TF was seen when it was considered as a continuous variable (p = 0.001). When using cut-off values of the 25^th^, 50^th^ and 75^th^ percentiles, TF in children where the household head had 1–3 years of education (Wald p = 0.440) and 4–6 years of education (Wald p = 0.538) was not different to the reference category of 0 years. The categorical variable was therefore compressed for increased power to a binary variable, with categories 0–6 years and 7+ years of household head education. In The Gambia, very few children had TF in households where the household head had at least one year of education and categorisation was made into 0 years and at least one year, to indicate some level of education (Table 3).

#### Multivariate analysis

In The Gambia, covariates added in turn after *a priori* inclusion of age and sex to the model, were ocular and nasal discharge, flies on the child's face, education of the household head (categorised as 0 years or ≥1 year) and time to primary water source. In Tanzania, the covariates added in turn were ocular and nasal discharge, flies on the child's face, being Amplicor positive, total household size, and education of the household head (categorised as 0–6 years or 7+ years).

The final multivariable regression model for each country demonstrated that being aged ≥1 year, ocular and nasal discharge, and no or reduced levels of household head education remained associated with increased risk of TF. These were true for both settings. In The Gambia, there was weak evidence that poor access to water was associated with TF. In Tanzania, after adjustment, observing flies on the face, being Amplicor positive, and an index of crowding (an increase in the number of children aged 0–5 years per household), were additionally related to an increase in TF (Table 4).

**Table 4:** Multivariate TF risk factor analyses in children aged 0–5 years in The Gambia and Tanzania.

		The Gambia		Tanzania	
Characteristic		OR (95% CI)	p-value^a^	OR (95% CI)	p-value^a^
**Child level**					
Total	-		-		
Age (years)	0	1		1	
	1	4.77 (2.38–9.58)	<0.001	4.26 (2.60–6.99)	<0.001
	2	5.98 (3.01–11.9)	<0.001	6.67 (4.05–11.1)	<0.001
	3	6.37 (3.22–12.6)	<0.001	10.0 (5.95–16.8)	<0.001
	4	7.20 (3.65–14.2)	<0.001	7.48 (4.49–12.5)	<0.001
	5	6.81 (3.41–19.6)	<0.001	5.37 (3.26–8.84)	<0.001
Sex	Female	1		1	
	Male	0.78 (0.60–1.02)	0.071	1.09 (0.87–1.36)	0.463
Ocular Discharge	No	1		1	
	Yes	4.16 (2.92–5.92)	<0.001	1.92 (1.32–2.80)	0.001
Nasal Discharge	No	1		1	
	Yes	1.63 (1.23–2.17)	0.001	1.53 (1.21–1.94)	<0.001
Flies on the face	No	-	-	1	
	Yes	-	-	1.68 (1.04–2.73)	0.035
Amplicor positive	No	-	-	1	<0.001
	Yes	-	-	14.07 (8.98–22.0)	
**Household level**					
Number of people/household^b^	Children 0–5	-	-	1.18 (1.02–1.36)	0.027
Household head education	Gm: NoneTz: 0–6 years	1		1	
	Gm: ≥1 yearTz: 7+ years	0.42 (0.16–1.09)	0.075	0.77 (0.60–0.98)	0.032
Time to water	<30 minutes	1		-	-
	≥30 minutes	1.50 (0.95–2.34)	0.079	-	-

TF = trachomatous inflammation, follicular; OR = Odds Ratio; CI = Confidence Interval; Analyses adjusted for variables included in final multivariable regression model.

a p-value from Wald test in multivariable regression analysis.

b variable modelled as continuous measures.

### Risk factors for ocular *C. trachomatis* infection

The low prevalence of Amplicor positives in The Gambia provided little power for formal risk factor analyses (Table 5). Chi-squared tests of association suggested that ocular discharge was a possible risk factor for an Amplicor positive result (p = 0.044) and that prevalence varied by district (p<0.001). In Tanzania, Amplicor positivity was associated in univariate analyses with being aged ≥1 year, having ocular or nasal discharge, flies on the child's face, lack of household head education, and poor access to water or a latrine (Table 5). In multivariate models, being Amplicor positive was only significantly related to being aged 2–5 years, having discharge, and a head of household educational level of less than 7 years, and possibly poor access to water (Table 5). Other factors were not related to evidence of *C. trachomatis* infection.

**Table 5:** Amplicor positive risk factor associations in children aged 0–5 years in The Gambia and Tanzania.

		The Gambia			Tanzania					
Characteristic		N	Amplicor positive, n (%)	p-value^a^	N	Amplicor positive, n (%)	Unadjusted OR (95% CI)	p-value^b^	Adjusted^c^ OR (95% CI)	p-value^d^
**Child level**										
Total	5036	39 (0.8)	-	3122	684 (21.9)	-	-	-	-	
Age (years)	0	792	4 (0.5)	0.791	550	61 (11.1)	1	<0.001	1	-
	1	805	9 (0.1)		468	76 (16.2)	1.96 (1.04–3.69)		1.70 (0.91–3.17)	0.095
	2	801	5 (0.6)		569	122 (21.4)	4.89 (2.59–9.25)		4.03 (2.17–7.51)	<0.001
	3	897	8 (0.9)		553	134 (24.2)	6.07 (3.25–11.3)		5.54 (3.00–10.2)	<0.001
	4	824	6 (0.7)		504	156 (31.0)	12.0 (6.03–23.9)		11.2 (5.71–22.1)	<0.001
	5	917	7 (0.8)		478	135 (28.2)	0.79 (4.89–19.6)		8.89 (4.56–17.7)	<0.001
Sex	Female	2442	19 (0.8)	0.977	1539	355 (23.1)	1	0.260	1	0.293
	Male	2594	20 (0.8)		1583	329 (20.8)	0.85 (0.65–1.12)		0.85 (0.62–1.16)	
Ocular Discharge	No	4578	32 (0.7)	0.044	2810	579 (20.6)	1	<0.001	1	<0.001
	Yes	2594	7 (1.6)		301	101 (33.6)	2.39 (1.54–3.71)		2.56 (1.54–4.28)	
Nasal Discharge	No	3370	24 (0.7)	0.460	1579	311 (19.5)	1		1	0.036
	Yes	1654	15 (0.9)		1513	369 (24.4)	1.55 (1.16–2.07)	0.002	1.42 (1.02–1.97)	
Flies on the face	No	4279	33 (0.8)	0.907	2937	622 (21.2)	1		-	-
	Yes	739	6 (0.8)		173	58 (33.5)	1.86 (1.04–3.33)	0.035	-	-
**Household level**										
Number of people/household	All residents	-	-	0.134^e^	-	-	1.01 (0.95–1.08)	0.676	-	-
	Children 0–5	-	-	0.140^e^	-	-	1.15 (0.96–1.38)	0.124	-	-
Household head education^f^	Continuous	-	-	-	-	-	0.92 (0.88–0.97)	0.001	-	-
	0 / 0-6 years	4736	36 (0.8)	0.646	1634	405 (24.8)	1		1	<0.001
	≥1 / 7+ years	300	3 (1.0)		1486	278 (18.7)	0.55 (0.40–0.76)	<0.001	0.51 (0.35–0.74)	
Time to water	<30 minutes	4323	32 (0.7)	0.495	720	122 (16.9)	1	0.017	1	0.090
	≥30 minutes	713	7 (1.0)		2401	562 (23.4)	1.74 (1.09–2.76)		1.58 (0.93–2.64)	
Latrine access	No	387	5 (1.3)	0.227	1066	263 (24.7)	1	0.048	-	-
	Yes	4649	34 (0.7)		2054	421 (20.5)	0.72 (0.51–1.00)		-	-
Health education program	No	3343	28 (0.8)	0.359	1803	413 (22.9)	1	0.153	-	-
	Yes	1669	10 (0.6)		1159	227 (19.6)	0.78 (0.55–1.10)		-	-

OR = Odds Ratio; CI = Confidence Interval.

a p-value from chi-squared test;

b p-value from likelihood ratio test comparing random effects logistic regression models adjusting for between-household and between-cluster variation with and without characteristic of interest;

c Adjusted for variables included in final multivariable regression model as shown;

d p-value from Wald test in multivariable regression analysis;

e Wald p-value from logistic regression with robust standard errors;

f reference category for The Gambia = 0 years and comparison category is ≥1 year; reference category for Tanzania = 0–6 years and comparison category is 7+ years. Continuous education measure centred prior to regression modelling.

## Discussion

As expected, the TF and Amplicor positive prevalences were lower in the Gambian than in the Tanzanian sample, with pre-treatment prevalences similar to reported national estimates [5], [6]. The Gambia and Tanzania have different trachoma control profiles. The Gambia is the smallest country on the African continent. Its National Eye Care Programme (NECP) has been in operation since 1986, which covered the whole country by 1996 [17]. There is also evidence of trachoma decline in the absence of specific control interventions but associated with improvements in sanitation, water supply, education, and access to health care in the villages [18]. In contrast, the national trachoma control programme in Tanzania started in 1999, with SAFE incorporated into the district health plan of 12 of the 50 trachoma-endemic districts by 2008 [19]. Mass azithromycin treatment in The Gambia began in November 2007, whereas in Tanzania distribution programmes started in 1999.

The risk factor data suggested similarities between the two countries in that TF was related to signs of an unclean face (ocular or nasal discharge), the level of household head education, and the child being aged ≥1 year. In the multivariate models, the association with access to water in The Gambia was weak and there was no association in Tanzania, suggesting that univariate associations were driven by confounding with education or facial cleanliness markers. Interestingly, there were additional risk factors for TF in Tanzania: being Amplicor positive, an index of crowding (the number of children per household), and flies on the children's faces. This may reflect that transmission of *C. trachomatis* is easier and more frequent in Tanzania, though we did not find an association between evidence of being Amplicor positive and crowding or flies on the face. It may be that through crowded conditions and exposure to flies, there is continual stimulation of the follicular immune response because of unhygienic living conditions, or the presence of a low level of inoculum that either does not lead to productive infection or a short lived infection.

Some level of education for the household head was a protective factor for TF in both settings. In The Gambia, few household heads had formal education, as has previously been documented [20], whereas Tanzanian household head formal education was better. A higher education level, a possible proxy for higher socio-economic status, has been associated with reduced risk of trachoma in other studies [21], [22]. In Egypt, low service utilisation has been associated with low educational and socio-economic level [23], which may be an explanation for the protective effect afforded by a greater number of years of household head education.

Knowledge of a health education programme in the village was not associated with TF in either setting. A third of Tanzanian households reported knowing of a health education programme, which is similar to that previously reported in the same Tanzanian district [24]. However, 693 households had missing data for this variable, based on the households reporting “don't know” to the question. Thus, this may not be the most reliable method of collecting data on the presence of health education programmes in communities since recall may be poor. The proportion of households in The Gambia aware of a health education programme was similar to that in Tanzania, despite village health workers not being required to conduct village-level hygiene sessions as they are in Tanzania. In Tanzania, the households that responded “don't know” may have done so if they did not consider the hygiene promotion provided by village health workers as formal health education. Health education programmes have previously been associated with clean faces when examined in clinics, indicating the potential impact that these programmes can have on facial cleanliness [24]. However, there is evidence that health education alone does not result in effective behaviour change [25], [26].

Both ocular and nasal discharge were associated with increased risk of TF in both settings. Discharge has been closely associated with active trachoma in many studies, including those from Tanzania and The Gambia [20], [27], [28], [29], but the causal relationship remains unclear. Infected discharge may aid transmission via fingers, flies or fomites. Discharge may however also be the consequence of trachoma, as inflammation of the conjunctiva could result in discharge being produced [20], [24].

Less than 10% of children had ocular discharge or flies on their face in Tanzania, although nearly half had nasal discharge. The association between TF and flies in Tanzania accords with previous risk factor studies from this country [13], [29], [30], [31]. In The Gambia, the prevalence of flies on the face was higher (14.7%), ocular discharge was similar (8.8%), and nasal discharge was less (32.9%), than in Tanzania. In The Gambia, flies were not associated with TF after adjustment for age, sex, ocular and nasal discharge, indicating that the univariate effect of flies was explained by discharge. This is consistent with the finding by Emerson *et al.* in The Gambia that those with discharge had twice the fly-eye contacts of those without [32]. In Tanzania, the strength of the individual associations of ocular discharge, nasal discharge and flies on the face with TF was slightly weaker in the multivariate model than in the univariate model. In particular, the association with flies showed only some evidence of an association once adjusted for ocular and nasal discharge, indicating that some of the univariate association was explained by the presence of discharge.

If the presence of either nasal or ocular discharge is combined into a single sign of an unclean face, then the prevalence of unclean faces in the sentinel children in Tanzania, 50.9%, was greater than in The Gambia, 37.5%, perhaps reflective of the higher rate of trachoma and infection in the Tanzanian setting. Thus, despite similar awareness of health education, The Gambia appears to have better facial cleanliness. However, in our study, facial status was recorded at the time of examination and could therefore reflect parents cleaning their children's faces before taking them for screening, leading to an under-estimate [24].

Latrine access was not associated with TF in either country. In Tanzania, household latrine access was 64.8%, indicating that latrine provision at these levels may be sufficient. The provision of latrines is expected to result in a reduction in flies because *Musca sorbens* (the putative trachoma vector) has been seen to preferentially breed in exposed human faeces [33], but is not found exiting latrines [34]. Latrine access in The Gambia was 89.6%, and the importance of latrine access has previously been demonstrated in this setting [35]. However, access to a latrine does not necessarily mean the latrine is used [36]. Previously in the same Tanzanian district of Kongwa, Taylor *et al.* found an association between lack of latrine access and TI. They also noted that only 59% of households had access to a latrine, and 23.4% of these were non-functional [37]. In a case-control study in the same Tanzanian district, Montgomery *et al.* observed that latrine use was greater in households that were trachoma-free than households containing a case of active trachoma, and this association remained after adjustment for potential confounders [22]. Thus, measurement of latrine use, rather than latrine access, may be a more valuable marker of the effect of latrines on trachoma prevalence.

In The Gambia, a primary water source more than 30 minutes away was weakly associated with increased risk of TF. Interestingly, time to fetch water was not a risk factor for TF in Tanzania. Poor access to water was recently not reported as a risk factor for active trachoma in a comparable study area of The Gambia [20], whereas in Tanzania it is often an independent risk factor for active trachoma [30], [38]. Montgomery *et al.* found that there was an unadjusted association between households with worse access to water and having a case of active trachoma [22]. Access to water in this district has not changed in the past 20 years, as West *et al.* (1989) documented that 79.8% of children came from households more than 30 minutes away from the water source [39], which is similar to the 76.9% found in our study. The lack of an association in Tanzania despite no apparent change in access to water indicates that interventions other than water supply improvements may now be more important for trachoma control in this area of Tanzania.

However, as with latrine access and use, access to water does not necessarily correlate with behaviour change. In the study by West *et al.* (1989), although there was a greater risk of having active trachoma and an unclean face with increased distance to water, these outcomes were not associated with the estimated amount of water brought into the household [39]. In a Gambian study, families with active trachoma were likely to use less water per person per day to wash children than families with no cases of active trachoma, after adjustment for family size, distance to water and socio-economic indicators [40]. If it is assumed that discharge is a causal factor in trachoma transmission, it is possible that a combination of improved water access and community health education may lead to cleaner faces and a consequent decline in trachoma. However, as previously noted, interpretation of clean face data is difficult, and a decline in unclean faces may not correlate with a decline in trachoma.

In Tanzania only, an increased risk of TF was associated with an increase in the number of children per household. In Tanzania, West *et al.* similarly found an association between the number of children per household and active trachoma [39], and in The Gambia Bailey *et al.* observed that an increase in the number of people per bedroom was associated with active trachoma [41]. The number of children in the household is used as a marker for crowding, but the two are not necessarily correlated, as a few people may live in a small house and many people may live in a large house. A study in Mali showed that although increased risk of active trachoma was associated with bedrooms in which there were more than 4 people, it was also associated with households containing less than 10 people compared with more than 10 [21]. Without an understanding of the household physical space, it is not possible to determine whether a larger number of people is a true marker of crowding.

Risk factors for evidence of infection differed between the countries, largely due to the relative absence of Amplicor positives in The Gambia and the associated lack of power to observe associations. In Tanzania, where active trachoma and being Amplicor positive were well associated, risk factors for TF and Amplicor positivity were similar, although the environmental factors of crowding and flies on the face were not related to being Amplicor positive. In The Gambia, since ocular discharge was the only factor associated with evidence of infection, it may be the key infection transmission route at this low endemicity. We have previously suggested that child-level factors should be the main target for control interventions in this setting [20], and that targeting children may eliminate the last reservoirs of infection.

As with any risk factor study reliant on a questionnaire, the validity of the data is prone to responder bias. The questionnaires were designed to be simple and appropriate for both countries for logistic and comparability reasons. We therefore asked a limited number of questions and did not support these with observational data, such as evidence of water or latrine use, which may be better measures of these trachoma risk factors [22], [40]. The major limitation of cross-sectional risk factor questionnaires is the inability to determine causality. Thus, although active trachoma and infection were both associated with ocular discharge in both countries, this does not necessarily mean that ensuring the absence of ocular discharge (such as through hygiene promotion) will aid trachoma control. Furthermore, determination of an unclean face may have been affected by the children's faces being examined at the time of screening, rather than in their household setting [24].

In the Gambian districts of Lower and Central Baddibu, the prevalence of clinical signs appears to have decreased in the absence of mass azithromycin treatment within the two years preceding this study. Only one village was previously mass treated in April 2006. The overall TF prevalence of 6.7% observed in this study is lower than the prevalence of 10.7% found in children aged 1–9 years in a survey conducted in 2006 [5]. In 2006, the prevalence of TF in 0–5 year-olds was 19.2% (19/99) in Lower Baddibu and 25.0% (23/92) in Central Baddibu, whereas the districts' respective prevalences in the same age-group are now 9.2% and 10.1%. This is not surprising as the NECP has been active since 1986, and over 85% of households have access to a latrine and a water source within 30 minutes. The overall prevalence of Amplicor positives in The Gambia in this study was only 0.8%, similar to the 0.3% observed in 2006 [5]. These results indicate that there is very little ocular *C. trachomatis* infection remaining in The Gambia. This may explain the lower TF prevalence in those aged under 1 year (1.6%) compared with the older age groups who have a prevalence exceeding 6%. However, the lower prevalence in the younger age group may be because they have less exposure, or the follicular signs of trachoma only appear after repeated or persistent infections and are therefore rare in children aged less than 1 year. The lower prevalence may also be due to the lack of ability to mount a follicular response to organism in very young children. In older children, there may also be some factor other than *C. trachomatis* infection causing follicles to appear, such as other organisms like Moraxella and adenovirus [7].

The difference between the two countries is further accentuated by the association between presence of clinical signs and evidence of infection. In The Gambia, the association was poor with only 0.9% of those with active trachoma being infected. In contrast, 38.9% of Tanzanian children with disease were Amplicor positive. Interestingly, of the few cases with evidence of infection in The Gambia, the majority did not fulfil the WHO criteria for active trachoma. This could indicate that there was contamination of samples or Amplicor false-positives. However, only one (0.4%) air control was positive, the laboratory passed its standardisation with the UCSF laboratory, and there were only 39 (0.8%) Amplicor positives in total, indicating that the risk of contamination and false-positives was low. The association in Tanzania was more conventional, with most Amplicor positive individuals being classified as also having active trachoma. Disease in Tanzania was more severe than in The Gambia with a larger proportion of children with clinical signs having TI, and these were more likely to be Amplicor positive. As seen in other studies, the prevalence of infection is associated with disease severity [10], [42], [43], [44]. It is likely that the distribution of mass treatment in Tanzania might increase the disconnect between infection and disease, as infection rapidly declines but clinical signs of trachoma persist [7], [11], [45], [46].

### Conclusion

In summary, this study showed that despite different prevalences of active trachoma and evidence of infection between the Tanzanian and Gambian study sites, the risk factors for TF were similar. The risk factors for being Amplicor positive in Tanzania were similar to those for TF, whereas in The Gambia, only ocular discharge was associated with evidence of *C. trachomatis* DNA, suggesting that at this low endemicity, this may be the most important risk factor. The lack of an association between being Amplicor positive and having TF in The Gambia highlights the poor correlation between the presence of trachoma clinical signs and evidence of *C. trachomatis* infection in this setting.

## Supporting Information

Checklist S1 STROBE Checklist
(0.16 MB RTF)
